# The inhibitory activity of herbal medicines on the keys enzymes and steps related to carbohydrate and lipid digestion

**DOI:** 10.1186/s12906-016-1424-2

**Published:** 2016-11-04

**Authors:** Weerachat Sompong, Nuttapat Muangngam, Artitaya Kongpatpharnich, Chadakarn Manacharoenlarp, Chanatkarn Amorworasin, Tanyawan Suantawee, Thavaree Thilavech, Sirichai Adisakwattana

**Affiliations:** 1 Department of Nutrition and Dietetics, Faculty of Allied Health Sciences, Chulalongkorn University, Bangkok, 10330 Thailand; 2 Program in Nutrition and Dietetics, Department of Nutrition and Dietetics, Faculty of Allied Health Sciences, Chulalongkorn University, Bangkok, 10330 Thailand; 3 Program in Biomedical Sciences, Graduate School, Chulalongkorn University, Bangkok, 10330 Thailand

**Keywords:** Herbal medicines, Antihyperglycemia, Antihyperlipidemia, Antioxidant

## Abstract

**Background:**

Obesity and overweight are consistently associated with metabolic disorders including hyperglycemia and hyperlipidemia. Herbal medicines have been currently suggested as an alternative source of potentially useful antihyperglycemic, antihyperlipidemic, antioxidant activities. The objective of this study was to assess the in vitro inhibitory effects of eleven herbal medicines on carbohydrate and lipid digestive enzymes and the key steps of lipid digestion including the inhibition of micelle formation and the ability to bind bile acid. In addition, antioxidant activity of herbal medicines was also investigated.

**Methods:**

α-Glucosidase, pancreatic α-amylase, pancreatic lipase, and pancreatic cholesterol esterase inhibitory activities of aqueous extract of herbal medicines were measured using the enzymatic colorimetric assay. The formation of cholesterol micelles was determined using the cholesterol assay kit. The bile acid binding was measured using the colorimetric assay. Antioxidant activities were assessed by using four methods including Trolox equivalent antioxidant capacity (TEAC), oxygen radical absorbance capacity ORAC), superoxide radical scavenging activity (SRSA), and hydroxyl radical scavenging activity (HRSA).

**Results:**

The extracts (1 mg/mL) markedly inhibited intestinal maltase (5.16 − 44.33 %), sucrase (1.25–45.86 %), pancreatic α-amylase (1.75–12.53 %), pancreatic lipase (21.42–85.93 %), and pancreatic cholesterol esterase (2.92–53.35 %). The results showed that all extracts exhibited the inhibitory activity against pancreatic lipase with the IC_50_ values ranging from 0.015 to 4.259 mg/mL. In addition, the incorporation of cholesterol into micelles was inhibited by the extracts (6.64–33.74 %). The extracts also bound glycodeoxycholic acid (9.9–15.08 %), taurodeoxycholic acid (12.55–18.18 %), and taurocholic acid (11.91 − 18.4 %). Furthermore, the extracts possessed various antioxidant activities including the TEAC values (0.50 − 4.70 μmol trolox/mg dried extract), the ORAC values (9.14–44.41 μmol trolox/mg dried extract), the SRSA (0.31 − 18.82 mg trolox/mg dried extract), and the HRSA (0.05–2.29 mg trolox/mg dried extract). The findings indicated that *Syzygium aromaticum*, *Phyllanthus amarus*, *Thunbergia laurifolia* were the effective promising antihyperglycemic and antihyperlipidemic agents. Statistical analysis demonstrated strong positive significant correlations between the contents of phenolic compounds and % inhibition of pancreatic lipase (*r* = 0.885, *p* < 0.001), % inhibition of pancreatic cholesterol esterase (*r* = 0.761, *p* < 0.001), and the TEAC value (*r* = 0.840, *p* < 0.001). Furthermore, there was a strongly positive correlation between the TEAC value and % inhibition of pancreatic cholesterol esterase (*r* = 0.851, *p* < 0.001) and % inhibition of pancreatic lipase (*r* = 0.755, *p* < 0.001).

**Conclusions:**

Three herbal medicines including *Syzygium aromaticum, Thunbergia laurifolia, and Phyllanthus amarus* markedly inhibited the intestinal α-glucosidase, pancreatic α-amylase, pancreatic lipase, and pancreatic cholesterol esterase. They also reduced formation of cholesterol micelle and bound bile acid. The findings indicate that these herbal medicines might be a promising agent for antihyperglycemic, antihyperlipidemic, and antioxidant activities.

## Background

The prevalence of obesity and overweight has dramatically increased worldwide due to a modern lifestyle and an increase of consumption of high-fat and high-carbohydrate diets [1]. Obesity and overweight are consistently associated with the elevation of blood glucose and lipid levels. Hyperglycemia and hyperlipidemia are major risk factors for developing chronic diseases such as diabetes mellitus and cardiovascular diseases [1]. One of the interesting strategies for the prevention of obesity is to reduce or slow dietary carbohydrate and fat digestion and absorption in the small intestine [2, 3]. The results from this action cause attenuated rises in postprandial hyperglycemia, hypertriacylglycerolemia and hypercholesterolemia, consequently, reduced risks of the progression of cardiovascular diseases and diabetes and its complications [4, 5]. Additionally, consumption of carbohydrate- and fat-enriched foods results in a significant increase in postprandial glucose and oxidative stress by the formation of reactive oxygen species through several biochemical pathways [6]. Current evidence supports that the well-known α-glucosidase inhibitors (acarbose and voglibose) and pancreatic lipase inhibitor (orlistat) are clinically used for treatment of hyperglycemia and hyperlipidemia [7, 8]. In overweight and obese people, the suppression of postprandial glucose and lipid levels may reduce the incidence of developing diabetes and its complications [9]. However, they have been reported that use of these inhibitors is associated with unpleasant gastrointestinal side effects such as abdominal pain, flatulence, meteorism, and diarrhea [10, 11].

Nowadays, numerous herbal medicines have been reported as good sources of unique phytochemical compounds such as polyphenols and flavonoids. The scientists have investigated potential herbal medicines for the development of newer therapeutics for biologically active antioxidants, antihyperlipidemic and antihyperglycemic agents from natural resources, especially the reduction of carbohydrate and fat digestion and absorption [12, 13]. As described in Table 1, herbal medicines have been commonly used for the treatment of various diseases in the Ayurvedic system of Thai traditional medicine. Previous reports have shown the methylglyoxal-trapping abilities of herbal medicines and their inhibitory activities on the formation of protein glycation [14, 15]. Nevertheless, studies addressing the inhibition of these herbal medicines on the key enzymes and steps of carbohydrate and lipid digestions remain unknown. Therefore, the present investigation was to evaluate the effects of eleven herbal medicines on the intestinal α-glucosidases and pancreatic α-amylase. Moreover, the study was also investigated the inhibition of pancreatic lipase and pancreatic cholesterol esterase activities as well as inhibiting formation of cholesterol micellization, and bile acid binding. Finally, eleven herbal medicines were elucidated their antioxidant activities including Trolox equivalent antioxidant capacity (TEAC), oxygen radical absorbance capacity (ORAC), hydroxyl radical scavenging activity (HRSA), and superoxide radical scavenging activity (SRSA).

**Table 1:** The list of plants was used of this study

Plant samples				
Scientific name	Family	Used part	Bioactive compounds	Medicinal properties
*Rhinacanthus nasutus*	Acanthaceae	Leaves	Rhinacanthin [34]	Antidiabetes [35], antimicrobial [36], antiproliferative [34]
*Cissus quadrangularis*	Vitaceae	Aerial parts	Flavonoids, triterpenoids, tannins [37]	Antidiabetes [37], hepatoprotective [38], antiobesity [39], bone health [40]
*Syzygium aromaticum*	Myrtaceae	Buds	Eugenol (essential oil), flavonoids, aromatic hydroxy acids, tannins [41], triterpenoids [25]	Antioxidant [41], antiulcer [42], immunomodulatory [43], anticancer [44], antidiabetes [25]
*Acanthus ebracteatus*	Acanthaceae	Leaves	Megastigmane, benzoxazinoids [45]	Antimicrobial [45], antitumor [46]
*Thunbergia laurifolia*	Acanthaceae	Leaves	Rosmarinic acid [47], polyphenols, carotenoids, sterols [48]	Antiinflammatory [47], antimutagenic [49], antidiabetes [30], hepatoprotective [50],
*Phyllanthus amarus*	Euphorbiaceae	Aerial parts	Triterpenoids, lignans [51]	Immunosuppressive [52], antimicrobial, antiinflammatory [53], antioxidant [54]
*Cassia alata*	Leguminosae	Leaves	Antraquinones, flavonoids [55]	Antioxidant, antiinflammatory [56], antimicrobial [55]
*Pluchea indica*	Asteraceae	Aerial parts	Eudesmane, terpenoids, thiophene, lignins, flavonoids [57]	Antiinflammatory [57], antituberculosis [58], antiulcer [59]
*Cryptolepis buchanani*	Asclepiadaceae	Aerial parts	Nicotinoyl alkaloid, pyridine alkaloid [60]	Antiinflammatory [60]
*Derris scandens*	Fabaceae	Aerial parts	Isoflavone, isoscandinone, scandenins [61, 62]	Antiinflammatory [61], antidiabetes [62]
*Schefflera leucantha*	Araliaceae	Leaves	Steroids, terpenoids, flavonoids [63]	Antioxidant, antityrosinase, antimicrobial [63]

## Methods

### Chemicals

Gallic acid, rat intestinal acetone powder, porcine pancreatic α-amylase, 4-methylumbelliferone, glucose oxidase kits and 3,5-dinitrosalicylic acid *p*-nitrophenylbutylrate (*p*-NPB), oleic acid, phosphatidylcholine, glycodeoxycholic acid, taurodeoxycholic acid, taurocholic acid, porcine cholesterol esterase, porcine pancreatic lipase were purchased from Sigma-Aldrich Co. (St. Louis, MO, USA). Cholesterol test kits were purchased from HUMAN GmbH Co. (Wiesbaden, Germany). Total bile acid kit was purchased from Bio-Quant Co. (San Diego, CA, USA). All other chemical reagents used in this study were of analytical grade.

### Plant materials

The plants were purchased from a specific herbal drugstore, Bangkok, Thailand. The plant authentication was performed according to our previous report [15]. The extraction of herbal medicines was done according to a previous report [15]. Briefly, the plants (20 g) were boiled in distilled water (800 mL) for 3 h at 95 °C before filtered through Whatman No. 1 filter paper. Thereafter, the extraction was lyophilized with a freeze drier. The lyophilized powder was stored at 4 °C in a dark bottle until analysis. The powder of extracts (final concentration: 1 mg/mL) was resuspended in distilled water before experiments.

### Intestinal α-glucosidase inhibitory activity

The intestinal α-glucosidase inhibitory activity was determined according to a previous method [16]. Briefly, 100 mg of rat intestinal acetone powder was homogenized in 3 mL of 0.9 % NaCl solution. The solution was centrifuged at 12,000 *g* for 30 min and then subjected to assay. The crude enzyme solution was incubated with 25 mM maltose or 160 mM sucrose together with various extracts in 0.1 M phosphate buffer (PBS), pH 6.9. The reaction was incubated at 37 °C for 30 min (maltase assay) or 60 min (sucrase assay). Thereafter, the mixtures were suspended in boiling water for 10 min to stop the reaction. The concentrations of glucose released from the reaction mixtures were determined by glucose oxidase method with the absorbance at a wavelength of 450 nm. Intestinal maltase and sucrase inhibitory activity was expressed as the percentage inhibition using the following formula:

$$ \%\kern0.5em \mathrm{Inhibition}\kern0.5em =\frac{Ab{s}_{Control}- Ab{s}_{Sample}}{Ab{s}_{Control}}\times 100 $$

Acarbose was used as positive control in this study.

### Pancreatic α-amylase inhibitory activity

The pancreatic α-amylase inhibition assay was done according to a previous method [16]. The various concentrations of the extracts were incubated with solution containing porcine pancreatic α-amylase (3 units/mL), starch (1 g/L), and 0.1 M PBS, pH 6.9, for 10 min, the reaction was then stopped by 1 % dinitrosalicylic (DNS) reagent (3,5-dinitrosalicylic acid, 0.2 % phenol, 0.05 % Na_2_SO_3_ and 1 % NaOH in aqueous solution). After heating at 100 °C for 10 min, 40 % potassium sodium tartarate solution was added to the mixtures to stabilize the color. After cooling to room temperature in a cold water bath, the absorbance was measured at 540 nm using a microplate reader.

$$ \%\kern0.5em \mathrm{Inhibition}\kern0.5em =\frac{Ab{s}_{Control}- Ab{s}_{Sample}}{Ab{s}_{Control}}\times 100 $$

Where Abs_Control_ was the absorbance without sample, Abs_sample_ was the absorbance of sample extract. Acarbose was used as positive control in this study.

### Pancreatic lipase inhibition

Pancreatic lipase activity was done according to a previous method [17]. The sample solution was incubated with the pancreatic lipase solution (12.5 U/mL) and 0.05 mM oleate ester of fluorescent 4-methylumbelliferone (4MUO) solution in 0.1 M PBS, pH 6.9. After incubation at 37 °C for 20 min, the reaction was terminated by 0.1 M sodium citrate (pH 4.2). The amount of 4-MUO released by lipase was determined using a fluorescence spectrophotometer at an excitation wavelength of 320 nm and an emission wavelength of 450 nm. Orlistat was used as a positive control in this study.

### Pancreatic cholesterol esterase inhibition

The pancreatic cholesterol esterase inhibition was performed according a previously described method [17]. The extract was incubated with the mixtures containing 5.16 mM taurocholic acid, 0.2 mM *p*-NPB in 100 mM sodium phosphate buffer, 100 mM NaCl, pH 7.0. After incubation with porcine pancreatic cholesterol esterase (1 μg/mL) for 5 min at room temperature, the mixtures were measured the absorbance at the wavelength of 405 nm. Simvastatin was used as a positive control for this study.

### Cholesterol micellization

Artificial micelles were prepared according to a previously described method [17]. In brief, the mixtures (2 mM cholesterol, 1 mM oleic acid, and 2.4 mM phosphatidylcholine) were dissolved in methanol and dried under nitrogen before adding 15 mM PBS containing 6.6 mM taurocholate salt, at pH 7.4. The suspension was sonicated twice for 30 min using a sonicator. The micelle solution was incubated overnight at 37 °C. The extract or equivalent PBS as control were added to the mixed micelle solution and incubated for a further 2 h at 37 °C. The solution was then centrifuged at 16,000 rpm for 20 min. The supernatant was collected for the determination of cholesterol by using total cholesterol test kits. Gallic acid was used as a positive control.

### Bile acid binding

The bile acid binding assay was done according to a previous method [17]. Taurocholic acid, glycodeoxycholic acid and taurodeoxycholic acid were used as bile acid in this experiment. Briefly, the extract (1 mg/mL) was incubated with each bile acid (2 mM) containing in 0.1 M PBS, pH 7 at 37 °C for 90 min. The mixtures were filtered through 0.2 μm filter and the bile acid concentration was determined using a bile-acid analysis kit. Cholestyramine was used as a positive control in this study.

### Trolox equivalent antioxidant capacity (TEAC) assay

The TEAC assay was performed according to a previous publication [14]. The absorbance was measured at 595 after incubation using a spectrophotometer. The TEAC value was calculated from a standard curve using a Trolox. The TEAC value was expressed as micromole of Trolox equivalents per milligram of extract.

### Oxygen radical absorbance capacity (ORAC) assay

The ORAC assay was determined according to a previously described method by using 2,2’-azo-bis (2-amidinopropane) dihydrochloride (APPH), a free radical generator solution [14]. The fluorescence intensity at an excitation wavelength 485 nm and emission wavelength 535 nm was recorded every 2 min for 60 min. A standard curve was generated with a Trolox concentration range from 0 to 48 μM. The ORAC value was calculated as the area under the curve (AUC) and expressed as micromole of Trolox equivalents per milligram of extract.

### Hydroxyl radical scavenging activity (HRSA)

The HRSA was measured according to a previously described method by using the mixture of hydroxyl radical solution [14]. The absorbance was measured at 532 nm. The HRSA value was calculated from a standard curve using a Trolox. The HRSA value was expressed as milligram of Trolox equivalents per milligram of extract.

### Superoxide radical scavenging activity (SRSA)

The SRSA was measured according to a previously described publication by using the mixture of superoxide radical solution [14]. After incubation for 40 min at 37 °C, the absorbance was determined at 560 nm. The SRSA value was calculated from a standard curve using a Trolox. The SRSA value was expressed as milligram of Trolox equivalents per milligram of extract.

### Statistical analysis

Data were expressed as mean ± standard error of the mean of three replicate determinations. Pearson’s correlation analysis was used to determine the correlation between total phenolic content (TPC) and antioxidant activity, the percentage of inhibition of pancreatic lipase, and pancreatic cholesterol esterase (SPSS Statistics 17.0, SPSS Inc., Chicago, IL, USA). A value of *P* < 0.001 was considered to be statistically significant.

## Results

The results in Table 2 demonstrate the percentage inhibition of herbal medicines against the intestinal α-glucosidases (maltase and sucrase) and pancreatic α-amylase. At concentration of 1 mg/mL, herbal medicines markedly inhibited intestinal maltase, ranging from 5.16–44.33 %. Among eleven herbal extracts, *Syzygium aromaticum* and *Schefflera leucantha* were the highest and lowest effective intestinal maltase inhibitor, respectively. They slightly inhibited the intestinal sucrase with the percentage inhibition of 1.25–45.86 %. It was found that *Phyllanthus amarus* was the most effective inhibitor, whereas *Cissus quadrangularis* had the lowest potent intestinal sucrase inhibitor among those of extracts. It was noted that herbal medicines showed weak inhibition against pancreatic α-amylase (1.75–12.53 %). Furthermore, acarbose (0.005 mg/mL) markedly inhibited intestinal maltase and sucrase with 64.87 ± 0.32 % and 11.42 ± 2.49 %, respectively. At the concentration of 0.16 mg/mL, acarbose had pancreatic α-amylase inhibitory activity with 60.22 ± 2.90 %.

**Table 2:** % Inhibition of herbal medicines (1 mg/mL) against pancreatic α-amylase, the intestinal α-glucosidase (maltase and sucrase), pancreatic lipase, and pancreatic cholesterol esterase

Plant samples	% Inhibition				
	α-Pancreatic amylase	Maltase	Sucrase	Pancreatic lipase	Pancreatic cholesterol esterase
*Rhinacanthus nasutus*	N.I.	2.72 ± 0.93	6.10 ± 1.43	55.82 ± 4.34	15.06 ± 0.56
*Cissus quadrangularis*	2.98 ± 0.03	7.50 ± 1.12	3.99 ± 1.44	21.42 ± 4.36	12.79 ± 3.32
*Syzygium aromaticum*	N.I	44.33 ± 2.96	45.08 ± 2.22	85.93 ± 0.53	53.55 ± 1.07
*Acanthus ebracteatus*	N.I	11.72 ± 4.91	2.69 ± 0.41	29.64 ± 4.19	2.92 ± 1.05
*Thunbergia laurifolia*	11.40 ± 2.58	10.10 ± 0.49	16.62 ± 3.11	54.25 ± 3.00	25.39 ± 2.42
*Phyllanthus amarus*	12.53 ± 3.00	38.25 ± 0.60	48.49 ± 1.85	65.30 ± 0.47	20.04 ± 0.41
*Cassia alata*	6.80 ± 2.63	5.66 ± 0.58	1.25 ± 0.49	59.24 ± 1.37	18.14 ± 1.55
*Pluchea indica*	12.04 ± 1.80	30.31 ± 2.15	1.84 ± 0.20	50.94 ± 0.12	12.76 ± 1.46
*Cryptolepis buchanani*	N.I.	5.16 ± 0.90	4.30 ± 1.02	38.79 ± 1.85	24.72 ± 1.91
*Derris scandens*	1.75 ± 0.86	8.09 ± 0.87	4.30 ± 1.02	51.48 ± 0.26	10.30 ± 0.73
*Schefflera leucantha*	N.I.	N.I.	5.25 ± 2.01	38.51 ± 1.50	30.90 ± 2.45
Acarbose^a,b^	60.22 ± 2.90	64.87 ± 0.32	11.42 ± 2.49	-	-
Orlistat^c^	-	-	-	76.55 ± 2.72	-
Simvastatin^d^	-	-	-	-	51.01 ± 0.79

Results are represented as mean ± SEM (*n* = 3). *N.I*. = No inhibition. ^a^Acarbose at concentration 0.16 mg/mL for pancreatic α-amylase inhibition and ^b^acarbose at concentration 0.005 mg/mL for maltase and sucrase inhibition. ^c^Orlistst at concentration 0.063 mg/mL. ^d^Simvastatin at concentration 0.13 mg/mL

**Fig. 1:**
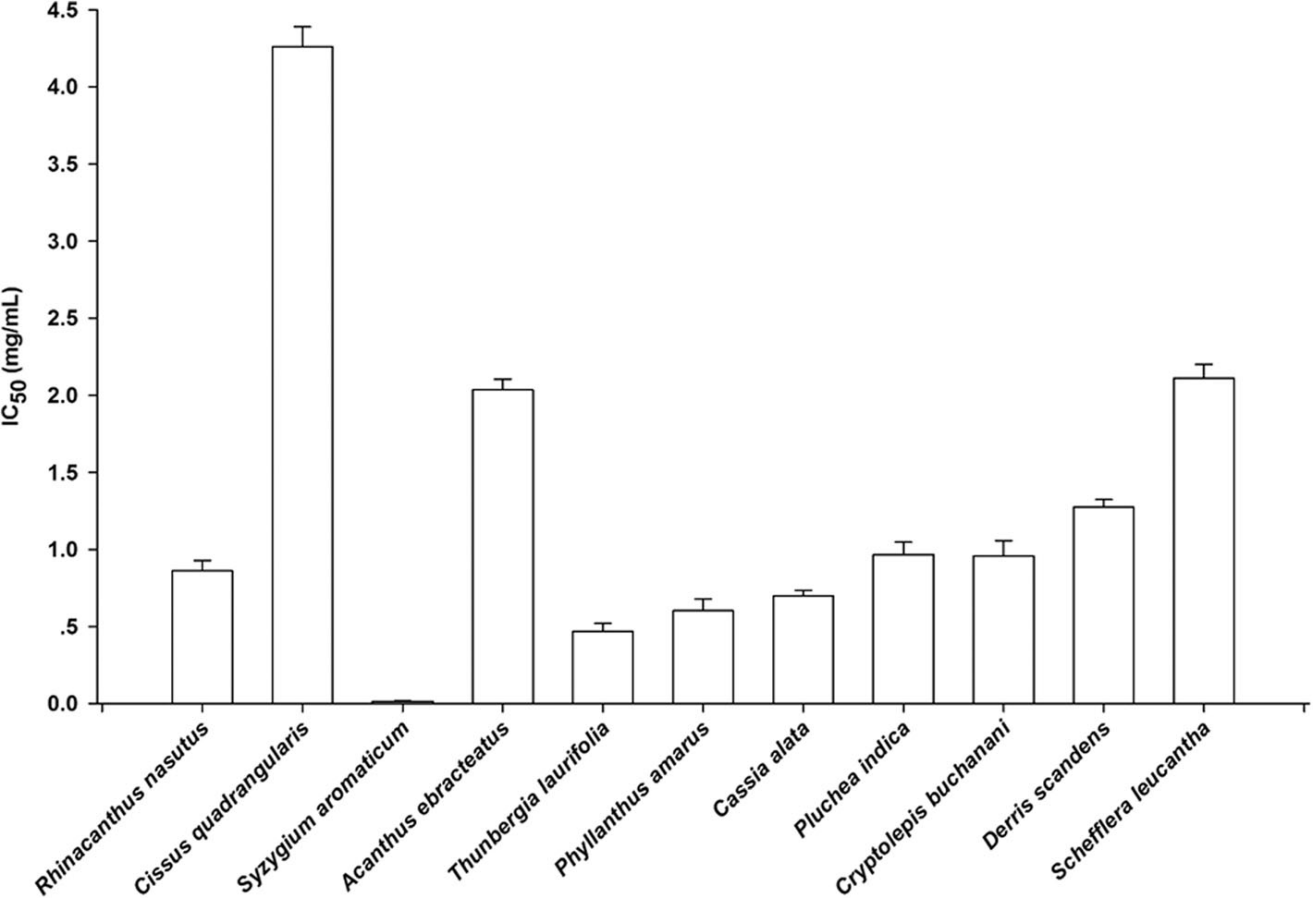
The IC_50_ values of herbal medicines against pancreatic lipase. The results are represented as mean ± SEM (*n* = 3)

The results of pancreatic lipase and cholesterol esterase inhibition by various herbal medicines (1 mg/mL) are summarized in Table 2. A variety of the tested plant extracts showed a strong inhibitory potential against pancreatic lipase with ranging from 21.42 to 85.93 %. The extracts were further investigated for their pancreatic lipase inhibitory effects at different concentrations. The results showed that all extracts markedly inhibited pancreatic lipase activity in a concentration-dependent manner. As shown in Fig. 1, *Syzygium aromaticum* was the most effective pancreatic lipase inhibitor with IC_50_ values of 0.015 ± 0.002 mg/mL, whereas *Cissus quadrangularis* was the lowest potent inhibitor among those of the extracts (IC_50_ = 4.259 ± 0.131 mg/mL). However, all extracts were less potent than that of orlistat on inhibition of pancreatic lipase (IC_50_ = 0.058 ± 0.005 mg/mL). At concentration of 1 mg/mL, *Syzygium aromaticum* markedly inhibited pancreatic cholesterol esterase activity with 53.35 ± 1.07 %, whereas other herbal medicines attenuated this enzyme activity with the range of 2.92–30.9 %. In addition, simvastatin had the percentage inhibition of 51.01 ± 0.79 % at the concentration of 0.13 mg/mL.

The results in Table 3 describe the percentage inhibition of herbal medicines (1 mg/mL) on cholesterol micellization. *Syzygium aromaticum, Phyllanthus amarus, Rhinacanthus nasutus, and Thunbergia laurifolia* slightly reduced the formation of cholesterol micellization with values of 2.29–33.74 %, whereas other herbal medicines had no inhibitory activity. Gallic acid (0.06 mg/mL) markedly inhibited the formation of cholesterol micelles about 32.48 ± 3.33 %. The percentage of bile acid binding by herbal medicines (1 mg/mL) is shown in Table 3. The results showed that *Phyllanthus amarus* demonstrated the highest binding of glycodeoxycholic acid and taurocholic acid with a degree of 15.08 % and 18.40 %, respectively. The highest binding of taurodeoxycholic acid was observed with *Syzygium aromaticum* (18.18 %). Additionally, cholestyramine (1 mg/mL) bound 55.07 ± 2.85 %, 36.86 ± 2.27 %, and 61.20 ± 1.44 % of glycodeoxycholic acid, taurocholic acid, and taurodeoxycholic acid, respectively.

**Table 3:** The effect of herbal medicines (1 mg/mL) on inhibition of cholesterol micellization and bile acid binding

Plant samples	% Bile acid binding			
	% Cholesterol micellization inhibition	Glycodeoxycholic acid	Taurocholic acid	Taurodeoxycholic acid
*Rhinacanthus nasutus*	2.29 ± 0.77	N.I.	N.I.	N.I.
*Cissus quadrangularis*	N.I.	N.I.	N.I.	N.I.
*Syzygium aromaticum*	18.43 ± 0.179	13.47 ± 1.41	18.40 ± 2.04	18.18 ± 1.74
*Acanthus ebracteatus*	N.I.	N.I.	N.I.	N.I.
*Thunbergia laurifolia*	6.64 ± 0.67	9.90 ± 1.01	11.91 ± 1.36	12.55 ± 2.34
*Phyllanthus amarus*	33.74 ± 0.23	15.08 ± 1.44	16.77 ± 1.74	13.36 ± 4.51
*Cassia alata*	N.I.	N.I.	N.I.	N.I.
*Pluchea indica*	N.I.	N.I.	N.I.	N.I.
*Cryptolepis buchanani*	N.I.	N.I.	N.I.	N.I.
*Derris scandens*	N.I.	2.05 ± 0.27	4.19 ± 2.61	5.70 ± 0.39
*Schefflera leucantha*	N.I.	N.I.	1.76 ± 0.75	3.17 ± 1.21
Gallic acid (0.06 mg/mL)	32.48 ± 3.33	-	-	-
Cholestyramine (1 mg/mL)	-	55.07 ± 2.58	36.86 ± 2.27	61.20 ± 1.44

Results are represented as mean ± SEM (*n* = 3). *N.I*. = No inhibition

Antioxidant activities of eleven herbal medicines are presented in Table 4. The results demonstrated that the extracts had various antioxidant activity including TEAC, ORAC, SRSA, and HRSA with the range of 0.50–4.70 μmol trolox/mg dried extract, 9.14–44.41 μmol trolox/mg dried extract, 0.31–18.82 mg trolox/mg dried extract, and 0.05–2.29 mg trolox/mg dried extract, respectively. Comparing with a previous study [14], it was found that the highest TEAC, ORAC, and SRSA were elicited by *Syzygium aromaticum*. It was observed that *Cissus quadrangularis* had the highest SRSA in comparison with other herbal medicines.

**Table 4:** Antioxidant activity of herbal medicines including TEAC, ORAC, HRSA and SRSA

Plant samples	Antioxidant activity			
	TEAC	ORAC	SRSA	HRSA
*Rhinacanthus nasutus*	0.92 ± 0.01	18.31 ± 0.07	5.31 ± 0.45	1.86 ± 0.02
*Cissus quadrangularis*	0.52 ± 0.01	9.14 ± 0.05	0.31 ± 0.03	2.29 ± 0.05
*Syzygium aromaticum*	4.70 ± 0.03^a^	31.21 ± 0.21^a^	18.82 ± 0.50^a^	0.15 ± 0.04^a^
*Acanthus ebracteatus*	0.50 ± 0.01	10.77 ± 0.23	9.47 ± 0.39	0.38 ± 0.01
*Thunbergia laurifolia*	1.07 ± 0.01	44.41 ± 0.53	14.39 ± 0.24	1.48 ± 0.08
*Phyllanthus amarus*	1.40 ± 0.01	19.70 ± 0.17	15.77 ± 0.45	0.06 ± 0.02
*Cassia alata*	0.69 ± 0.01	14.63 ± 0.13	4.89 ± 0.31	1.15 ± 0.04
*Pluchea indica*	1.61 ± 0.01	28.06 ± 0.19	19.38 ± 0.50	0.05 ± 0.01
*Cryptolepis buchanani*	1.01 ± 0.01	21.22 ± 0.05	10.30 ± 0.18	1.14 ± 0.01
*Derris scandens*	1.38 ± 0.01	18.21 ± 0.23	7.32 ± 0.24	1.46 ± 0.01
*Schefflera leucantha*	0.86 ± 0.02	19.61 ± 0.44	7.80 ± 0.42	0.59 ± 0.02

Data are expressed as mean ± S.E.M, *n* = 3. TEAC was expressed as micromole trolox/ mg dried extract. ORAC was expressed as micromole trolox/ mg dried extract. Hydroxyl radical scavenging activity (HRSA) was expressed as milligram trolox/mg dried extract. Superoxide radical scavenging activity (SRSA) was expressed as milligram trolox/mg dried extract. ^a^antioxidant activity of *Syzygium aromaticum* was previously reported by our publication [62]

The total phenolic content (TPC) of extracts was previously reported by our experiments [15] that have been used for the calculation of Pearson’s correlation coefficients in the present study. The Pearson’s correlation coefficients between the variables are presented in Table 5. There were strong positive significant correlation between the contents of phenolic compounds and % inhibition of pancreatic lipase (*r* = 0.885, *p* < 0.001), % inhibition of pancreatic cholesterol esterase (*r* = 0.761, *p* < 0.001), and the TEAC value (*r* = 0.840, *p* < 0.001). Furthermore, a strongly positive correlation was also found between the TEAC value and % inhibition of pancreatic cholesterol esterase (*r* = 0.851, *p* < 0.001) and % inhibition of pancreatic lipase (*r* = 0.755, *p* < 0.001). In contrast, the negative correlation was observed between the HRSA and the SRSA value.

**Table 5:** Pearson’s correlation analyses of total phenolic content (TPC), % inhibition of pancreatic lipase (PL) (1 mg/mL), % inhibition of pancreatic cholesterol esterase (Chol) (1 mg/mL), ORAC, TEAC, SRSA, and HRSA

	TPC	PL	Chol	ORAC	TEAC	SRSA	HRSA
TPC	-	0.885*	0.761*	0.386	0.840*	0.594	-0.441
PL	-	-	0.643	0.547	0.755*	0.648	-0.454
Chol	-	-	-	0.490	0.851*	0.389	-0.197
ORAC	-	-	-	-	0.459	0.698	-0.203
TEAC	-	-	-	-	-	0.642	-0.457
SRSA	-	-	-	-	-	-	-0.785*
HRSA	-	-	-	-	-	-	-

^*^Correlation is significant at *P* < 0.001

## Discussion

Herbal medicines remain one of the most alternative approaches for the prevention and management of chronic degenerative diseases such as type 2 diabetes and cardiovascular diseases [12, 13]. Consumption of carbohydrate and fat enriched foods causes a rapid rise in the blood glucose and lipid profiles. In this regards, inhibition of pancreatic α-amylase and α-glucosidase impedes the postprandial glucose excursion to enable overall smooth glucose profile. Recent evidence also supports that aggressive delaying dietary fat digestion and absorption is a new strategy for prevention of hyperlipidemia [18]. Inhibition of pancreatic lipase and pancreatic cholesterol esterase results in delaying the process of hydrolyzing dietary triglyceride and cholesterol esters, respectively [19, 20]. In meantime, binding bile acids by forming insoluble complexes and increasing their fecal excretion as well as the reduction of micelle formation have been referred to as possible mechanisms for lowering blood cholesterol level [21]. Additionally, postprandial oxidative stress has been linked with consumption of a meal rich in lipids and/or carbohydrates [22]. The present findings are important, particularly in view of antihyperglycemic and antihyperlipidemic mechanisms of eleven herbal medicines including the intestinal α-glucosidase, pancreatic α-amylase, pancreatic lipase and pancreatic cholesterol esterase inhibitory activities as well as bile acid binding and the inhibition of cholesterol micellization. Furthermore, the medicinal properties of the plants have been explored for their potential antioxidant. Among the plant extracts examined, three herbal medicines exhibit a high potential for antihyperglycemic and antihyperlipidemic activity including *Syzygium aromaticum, Thunbergia laurifolia* and *Phyllanthus amarus*.

There are several reports demonstrating antihyperglycemic and antihyperlipidemic activity of three herbal medicines. For example, clove (*Syzygium aromaticum*) bud powder reduced blood glucose level in a high fat-induced diabetic rat [23]. A previous study revealed that eugeniin, the phytochemical compounds isolated from aqueous methanol extraction was attributed with the inhibition of rat intestinal maltase [24]. The present investigation evaluated the inhibitory activity of pancreatic α-amylase and α-glucosidase by using water-solvent extraction. The extraction method differs from previous studies which may result in various biological activities. *Syzygium spp*-derived triterpenes oleanolic acid (OA) and maslinic acid (MA) ameliorated postprandial hyperglycemia in diabetic rats through the inhibition of intestinal α-glucosidase and α-amylase [25]. OA have been reported to inhibit α-glucosidase as uncompetitive inhibition. The inhibitory activity of OA may be due to the triterpenoid structure [26]. The aqueous extract of *Syzygium aromaticum* also inhibited fructose-induced protein glycation and oxidation [14]. Furthermore, the ethanolic extract of *Syzygium aromaticum* markedly reduced serum triglyceride and cholesterol in high-fat diet-induced obese mice through downregulation of adipogenic and lipogenic gene expression [27]. However, other mechanisms of antihyperlipidemic activity of *Syzygium aromaticum* have not been described. The methanolic extract of *Phyllanthus amarus* was reported to have hypoglycemic effect on alloxan-induced diabetic rats [28]. In addition, significant alterations in lipid profiles were attenuated in diabetic rats treated with *Phyllanthus amarus* aqueous extract [29]. Moreover, the 15-day-treatment of *Thunbergia laurifolia* decreased plasma glucose concentration in diabetic rats [30]. Taken together, the present findings might describe antihyperglycemic and antihyperlipidemic mechanisms of three herbal medicines in diabetic rats.

Several reports support that total phenolic compounds in the herbal medicines have the ability to inhibit pancreatic α-amylase, α-glucosidase, pancreatic lipase activity and pancreatic cholesterol esterase [12, 31] whereas they reduce the formation of cholesterol micelles and bile acid binding [31, 32]. Other studies published earlier also showed positive correlation between phenolic compounds and the antioxidant activity of plant extracts and pancreatic lipase inhibitory activity [33]. The present results demonstrate a significant positive correlation between phenolic contents and pancreatic lipase inhibition activity, pancreatic cholesterol esterase, and antioxidant activity (TEAC) which provide strong support that phenolic compounds are key agents for pancreatic lipase and pancreatic cholesterol esterase inhibition as well as antioxidant activity.

## Conclusions

Eleven herbal medicines markedly demonstrated antioxidant, antihyperglycemic, and antihyperlipidemic activities, in particular, *Syzygium aromaticum, Thunbergia laurifolia* and *Phyllanthus amarus*. Interestingly, the phenolic compounds in the promising herbal medicines exhibited strong correlation to the inhibitory activity against pancreatic lipase, pancreatic cholesterol esterase, and the TEAC values. These results indicate that these herbal medicines could be a natural source for antioxidant, antihyperglycemic, and antihyperlipidemic agents.

## Abbreviations

Chol: pancreatic cholesterol esterase
HRSA: Hydroxyl radical scavenging activity
ORAC: Oxygen radical absorbance capacity
PL: Pancreatic lipase
SRSA: Superoxide radical scavenging activity
TEAC: Trolox equivalent antioxidant capacity
